# Supplementary material for: The interactome of intact mitochondria by cross-linking mass spectrometry provides evidence for coexisting respiratory supercomplexes
Source: Mol Cell Proteomics. 2017 Dec 8;17(2):216–32. doi: 10.1074/mcp.RA117.000470 (PMC5795388; doi:10.1074/mcp.RA117.000470)
Supplement: Supplemental Data [file supp_RA117.000470_133922_0_supp_23978_fzffwf.zip › spectra_annotation/mito_DR_spectra_annotation/313-1-12-1-8-1.pdf]

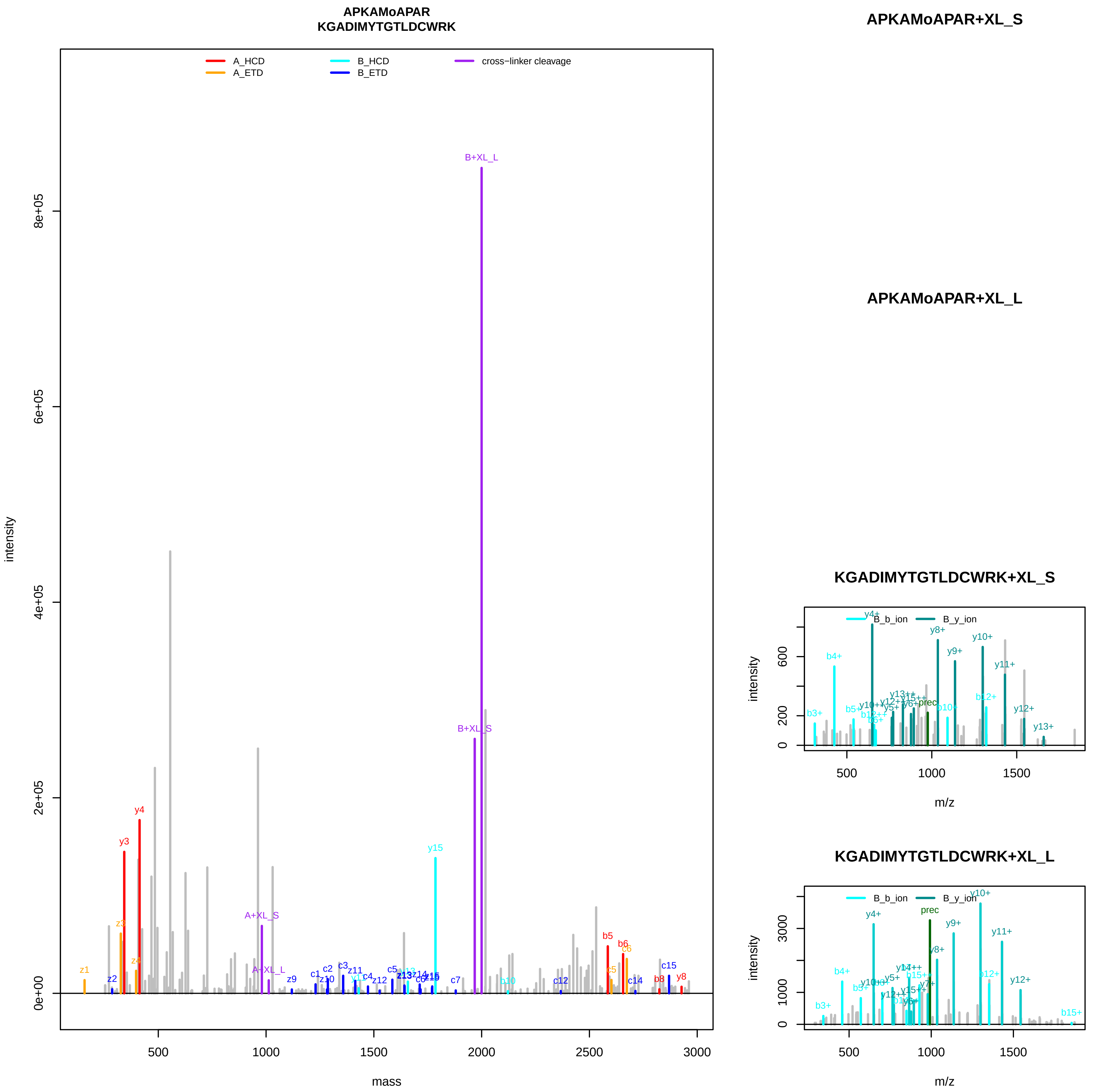
APKAMoAPAR
KGADIMYTGTLDCWRK
A_HCD
A_ETD
B_HCD
B_ETD
cross−linker cleavage
B+XL_L
B+XL_S
A+XL_S
A+XL_L
y4
y3
y15
b5
b6
c6
c5
b8
y8
c15
c14
c12
z1
z2
z3
z4
z9
c1
c2
c3
z10
z11
c4
z12
c7
intensity
mass
0e+00
2e+05
4e+05
6e+05
8e+05
500
1000
1500
2000
2500
3000
APKAMoAPAR+XL_S
APKAMoAPAR+XL_L
KGADIMYTGTLDCWRK+XL_S
B_b_ion
B_y_ion
y4+
y8+
y10+
y9+
y11+
y12+
y13+
b4+
b3+
b5+
b6+
b10+
b12+
prec
intensity
m/z
0
200
600
KGADIMYTGTLDCWRK+XL_L
B_b_ion
B_y_ion
y10+
y4+
prec
y9+
y8+
y11+
y12+
b4+
b3+
b5+
b12+
b15+
intensity
m/z
0
1000
3000
500
1000
1500
